# Robotic Platform: What It Does and Does Not Offer in Hernia Surgery

**DOI:** 10.3389/jaws.2024.12701

**Published:** 2024-02-14

**Authors:** Davide Lomanto, Lydia Tan, Sean Lee, Sujith Wijerathne

**Affiliations:** ^1^ Department of Surgery, Yong Loo Lin School of Medicine, National University of Singapore, Singapore, Singapore; ^2^ Department of General Surgery, Alexandra Hospital (National University Health System), Singapore, Singapore

**Keywords:** robotic surgery, hernia, inguinal hernia, ventral hernia, robotic platform

## Introduction

In the last few decades, surgery has become a fast-evolving field, where new technologies are introduced rapidly and spread across the various surgical sub-specialities. Among them, robotic surgery is one such technology, and its use is increasing, especially in the field of hernia surgery. Its fast adoption was to provide an alternative to open surgery by enhancing surgeons’ dexterity and ergonomics by utilising seven degrees of freedom and 3D vision, and also providing a significant advantage in restricted space like the pelvis and retromuscular plane, in which accurate dissection and suturing was a big challenge even for the more skilful surgeons. Ultimately, this resulted in an overall increase in the usage of minimally invasive hernia surgeries and related benefits for the patients [1]. While many surgeons today have embraced robotic surgery, there remains scepticism regarding cost-efficiency and long-term benefits [2].

### Technical and Ergonomic Advantages of Surgical Robotic Devices

Surgical robotic technology has advanced from mere passive robots used for retraction or holding instruments like rail-mounted devices or camera navigation to active robotic systems such as the Da Vinci Surgical system, Hugo Medtronic, Medicaid Hinotori, Versius CMR, and many more, which range of motion of surgical instruments enables high performance and enhanced precision for dissection, suturing the abdominal wall using a minimally invasive approach [3]. Furthermore, it eliminates physiological tremors and the fulcrum effect present in laparoscopic surgery. The wristed instruments of the robot provide several degrees of freedom to overcome the limitation of traditional laparoscopic instruments which often do not permit the tip of the instrument to reach the anterior abdominal wall [4] and allow suturing in challenging ergonomic positions. Thus, by utilising the robot, intracorporeal primary closure of fascial defect [5] and intraperitoneal placement of mesh are performed with greater ease and high success rate [6].

Surgeons who perform minimally invasive surgery are more likely to experience neck and shoulder pain compared to surgeons performing open surgery [7] because they tend to hold their neck in a static posture while simultaneously isometrically contracting the shoulder muscles in an abducted position during long operations [8]. On the contrary, in robotic surgery, the surgeon is seated at the console visualising the surgical field through a viewfinder while manipulating the hand controls and foot pedals to control the surgical instruments. Thus, with improved ergonomics, this reduces the risk of fatigue and backache [9]. Nevertheless, it is important to utilise proper posture and equipment setup as improper posture while performing robotic surgery can still lead to muscle aches and eye strain [10]. However, the option to set up your setting will minimise those health hazards.

### Reduced Complications

#### Inguinal Hernia Surgery

Multiple systematic reviews comparing robotic hernia surgery to laparoscopic or open surgery have shown reduced risks of complications in patients who underwent robotic surgery. De Angelis et al.‘s systematic review with pooled data analysis from 58 meta-analyses has shown that robotic inguinal hernia surgery was associated with a lower rate of hernia recurrences and conversion to open surgery compared to laparoscopic surgery [11]. Waitre et al. have reported reduced postoperative pain scores in robotic-assisted transabdominal preperitoneal repair compared to the conventional laparoscopic approach [12]. Yet, the RIVAL trial showed that for straightforward inguinal hernia, there was no clinical benefit using the robotic approach compared to the laparoscopic approach, and the robotic approach cost more, had longer operative time, and was associated with higher surgeon frustration with no ergonomic benefit to the surgeon [13].

With regards to robotic vs. open inguinal hernia, De Angelis et al.‘s systematic review has shown that robotic inguinal hernia repair was associated with fewer surgical site infections, less intraoperative blood loss, and shorter postoperative length of stay compared to open inguinal hernia repairs [11]. Bittner et al. showed significantly lower postoperative pain scores for laparoscopic and robotic inguinal hernia repair compared to open repairs [14]. Table 1 summarises the conclusions from the above studies discussed. Still, better-designed trials looking not only at patients’ perspectives but also at surgeons’ benefits are needed to fully resolve the issues. Significantly, robotic inguinal hernia repair has brought more surgeons to adopt the use of endolaparoscopic/robotic repair for inguinal hernia, a technique that was slowly adopted in the past.

**TABLE 1:** Studies analysing outcomes from robotic inguinal hernia repair compared with open and laparoscopic approaches.

Paper	Type of study	Conclusion
De Angelis et al. 2023 [11]	Systematic review	The robotic inguinal hernia was associated with lower rates of hernia recurrences and conversion to open surgery compared to the laparoscopic approach
		However, the robotic approach required a longer operative time
Waite et al. 2016 [12]	Retrospective study	Robotic TAPP repair had lower postoperative pain than laparoscopic TAPP repair
		However, robotic TAPPP required longer operative time and costs more
Prabhu et al. 2020 RIVAL trial [13]	Randomised Clinical Trial	Robotic inguinal hernia repair was associated with increased operative time, cost, and surgeon frustration without discernible ergonomic benefit for the surgeons, compared to laparoscopic repair
Bittner et al. 2018 [14]	Prospective study	Robotic inguinal hernia repair was associated with lower postoperative pain at 1 week, lesser activity disruption at 1 week, and shorter duration of analgesia use compared to open repair
		Robotic inguinal hernia repair had similar postoperative pain and activity restrictions at 1 week compared to laparoscopic repair

TAPP, transabdominal preperitoneal.

#### Ventral Hernia Surgery

**FIGURE 1:**
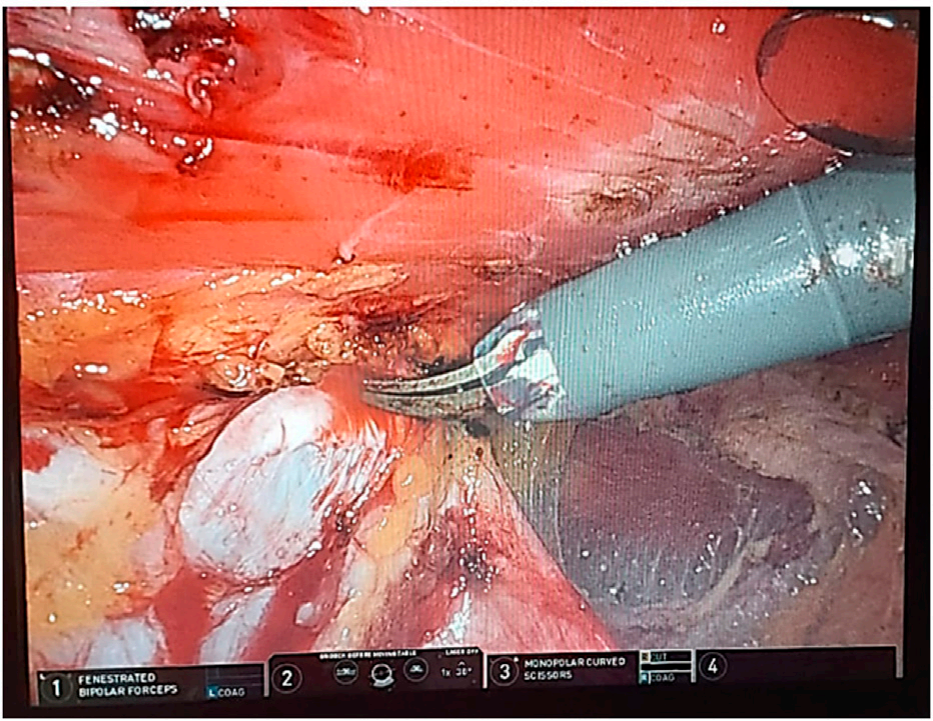
Depicts a cross-over to dissect both retro-rectus spaces.

**FIGURE 2:**
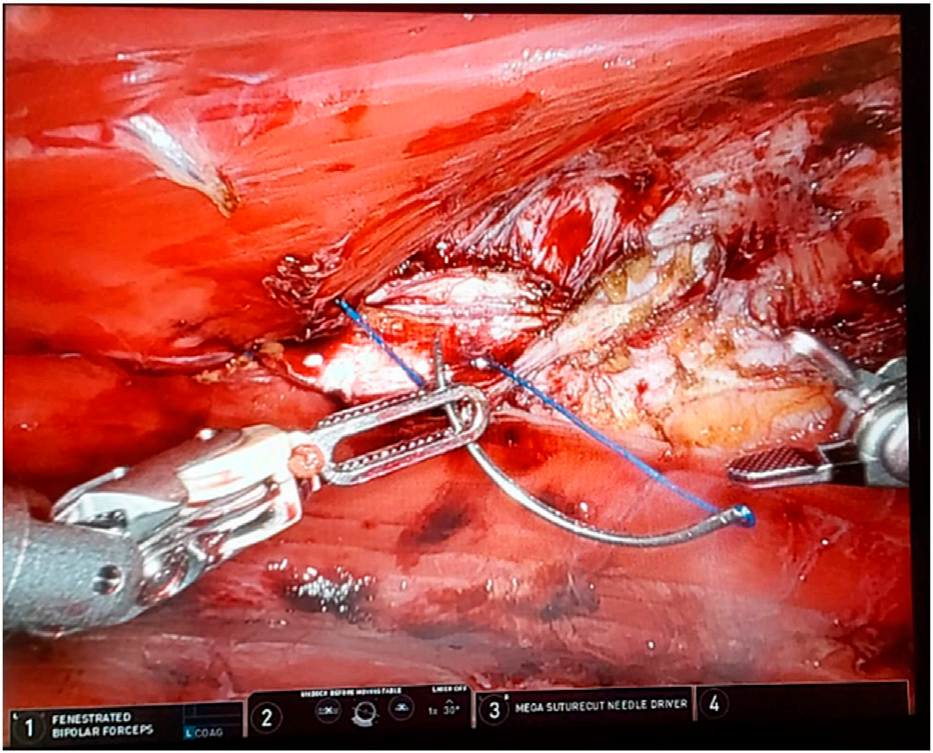
Depicts intraoperative suturing for the apposition of the rectus muscles.

**FIGURE 3:**
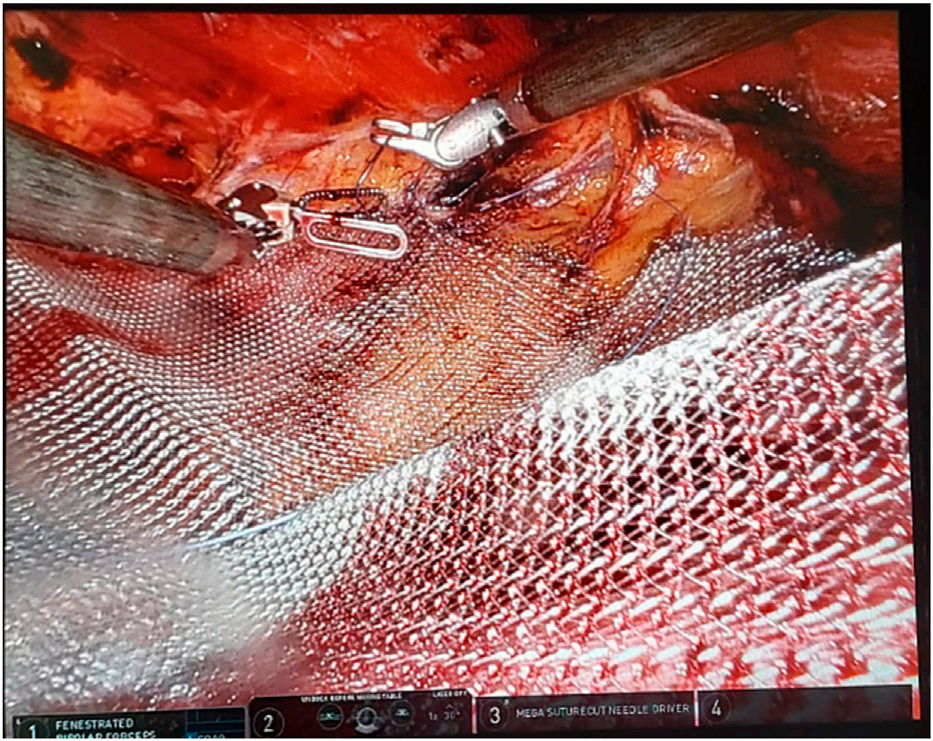
Depicts mesh placement in the retrorectus space.

In our institution, robotic extended totally extraperitoneal repair (eTEP) paraumbilical hernia and rectus diastasis repair is commonly performed. Figure 1 depicts a cross-over to dissect both retro-rectus spaces. Figure 2 depicts intraoperative suturing for the apposition of the rectus muscles. Figure 3 depicts mesh placement in the retrorectus space. The ease of intracorporeal suturing using a robot vs. laparoscopic approach facilitates surgery for larger hernias.

Yet, there have been varied results of robot-assisted hernia repair compared to laparoscopic and open repair. The challenges in ventral hernia repair, the rise of new technical approaches for ventral hernia repair, and the benefits of the robotic technology itself are all factors that have significantly contributed to the wide adoption and success of robotic ventral hernia repair, especially in the United State. The American database from Inpatient Sample found no difference in postoperative complications between robotic vs. laparoscopic ventral hernia repair [15], but the American Hernia Society Quality Collaborative showed fewer postoperative complications in robot-assisted intraperitoneal mesh placement (IPOM) vs. laparoscopic IPOM [16]. One of the main advantages resulting from utilising the robotic device in ventral hernia repair is that it can facilitate the placement of the mesh in the extraperitoneal space, avoiding possible related complications of an intraperitoneal mesh coming in contact with the bowel. Moreover, it has the possibility of approximating the recti muscles by closing the hernia defect and reconstituting the lines alba for better functionality of the abdominal wall. However, a systematic review showed that extra-peritoneal mesh does not appear to be superior to intraperitoneal mesh in the short term for minimally invasive ventral hernia repair [17]. Fascial closure was achieved in more than 90% of robotic ventral hernia cases compared to 50% of laparoscopic ventral hernia repairs. This was achieved largely with enhanced suturing in the robotic cases compared with tacks and sutures in the laparoscopic group [18]. Sutured closure of hernia defect reduces recurrence rates of bulging, pseudo-recurrence, and seroma formation [19]. Therefore, the robot proved to be useful in increasing the rate of achieved defect closure [20]. There were significantly smaller bowel injuries in the laparoscopic group compared with robotic ventral hernia repair [11, 21]. The length of postoperative stay was shorter for robotic ventral hernia compared to laparoscopic ventral hernia repair [22]. Yet, the PROVE-IT randomised clinical trial has shown comparable outcomes between laparoscopic and robotic ventral hernia mesh repair, and the increased operative time and cost of robotic ventral hernia surgery were not offset by a measurable clinical benefit [23]. Table 2 summarises the conclusions from the above studies.

**TABLE 2:** Studies analysing outcomes from robotic ventral hernia repair compared with open and laparoscopic approaches.

Paper	Type of study	Conclusion
De Angelis et al. 2023 [11]	Systematic review	Robotic ventral hernia was associated with lower rates of bowel injuries and conversion to open surgery compared to the laparoscopic approach
		The robotic ventral hernia was associated with lower postoperative complications, lower surgical site infections, lesser intraoperative blood loss, and shorter postoperative stay than open surgery
		However, the robotic approach required a longer operative time
Coakley et al. 2017 [15]	Retrospective study	Robotic ventral hernia repair was performed in older patients with more chronic conditions and cost more compared to the laparoscopic approach
Prabhu et al. 2017 [16]	Prospective study	Robotic IPOM was associated with a lower risk of surgical site infections and lower median length of stay than laparoscopic IPOM.
		However, operative time was longer in robotic IPOM than laparoscopic approach
Walker et al. 2018 [20]	Retrospective study	Robotic ventral hernia repair was associated with a decreased incidence of recurrence and surgical site infection compared to the laparoscopic approach
		The robotic approach was associated with increased primary fascial closure than the laparoscopic approach
Alfredo et al. 2018 [21]	Prospective study	Robotic ventral hernia repair was associated with a shorter length of stay than the open approach, but no significant differences were found for surgical site infections and 30-day readmission
Henriksen et al. 2019 [22]	Metanalysis	Robotic ventral hernia repair was associated with a shorter length of stay, but longer operative duration than the open approach
		No significant differences in postoperative complications
Petro et al. 2021 PROVE-IT [23]	Randomised Clinical Trial	No significant differences in postoperative pain, quality of life, and length of hospital stay between robotic and laparoscopic ventral hernia repair

IPOM, intraperitoneal mesh placement.

### Surgical Robot in Transversus Abdominis Release (Robo-TAR)

As stated, Surgical Robotics not only allows retromuscular ventral hernia repair but also enables abdominal wall reconstruction and extraperitoneal mesh placement that was previously only possible with open repair by reducing wound morbidity [24], with shorter postoperative stay [25]. Overall, robotic devices also allow for additional procedures like posterior component separation and transverses abdomen release to allow the closure of large abdominal defects that was possible only with the open approach. Despite the increased mean operative time for robotic TAR compared to open TAR repair, mean blood loss was lower in patients who underwent robotic TAR [19]. A systematic review showed that there were no statistical differences between robotic vs. open TAR repair in terms of re-operation rates, surgical site infections, and readmissions [26]. Table 3 summarises the above studies on robotic TAR versus the open approach.

**TABLE 3:** Studies analysing outcomes from robotic TAR compared with the open approach.

Paper	Type of study	Conclusion
Warren et al. 2017 [24]	Prospective study	Robotic ventral hernia was associated with increased rates of fascial closure and extraperitoneal mesh placement compared with the laparoscopic approach in which mesh was placed intraperitoneally
		Longer operative duration for the robotic approach compared with the laparoscopic approach
		The robotic approach was associated with increased seroma than the laparoscopic approach, but surgical site infection rates and narcotic requirements were similar
		The length of postoperative hospital stay was shorter with the robotic approach
		Direct hospital costs were similar between the robotic and laparoscopic approaches
Dewulf et al. 2022 [25]	Prospective study	Robotic TAR was associated with shorter postoperative stays and lower surgical site infections than open TAR.
		However, there were similar reoperation and recurrence rates between robotic and open approaches
Martin-del-Campo et al. 2017 [19]	Prospective study	Open TAR was performed for patients with more comorbidities compared to robotic TAR. Average operative duration was longer in robotic TAR, but average blood loss was lower, compared to open TAR. No differences in surgical site infections between the two approaches
		The length of postoperative stay was shorter for robotic TAR compared to the open approach
Bracale et al. 2021 [26]	Metanalysis	Robotic TAR was associated with a lower risk of complications and shorter postoperative stay, but longer operative duration compared to open TAR.
		There were no differences in rates of surgical site infections, readmission, and reoperation rates

TAR, transversus abdominus release.

### Disadvantages of Surgical Robotic Devices

Operative time was significantly longer for robotic hernia surgery compared with the open and laparoscopic approach [11, 27]. The time needed to dock the robot could have contributed to the longer procedure time in robotic surgery. There is currently inadequate evidence to show that the higher usage of the robot in experienced centres results in a lower docking time that may translate to comparable operative duration with laparoscopic surgery [11].

A significant factor to consider is the high cost of investing in the robotic device coupled with the cost of annual maintenance and per-case utilisation [27]. Further studies are needed to justify the cost-effectiveness of robot use in hernia surgery. These studies may consider focusing on quantifying cost savings from the reduced length of stay, and lower rate of postoperative complications juxtaposed to the increased cost of dedicated operating theatre, trained nursing staff, and cost of equipping healthcare professionals to utilise the robot effectively [28].

Specialised training can be time-consuming and may not be readily available in certain medical institutions. Apart from training surgeons to use the robotic consoles, training of adept assistants is required to reduce robot docking time and effectively facilitate surgery [29].

However, a few single-centre series have shown that robot-assisted transabdominal preperitoneal, total extraperitoneal (TEP), and single-site TEP repairs were feasible without a high-learning curve for a laparoscopic surgeon [12].

Lastly, the availability of robotic surgery may be limited in certain geographic locations. Thus, fewer patients are offered robotic surgery, which in turn results in the surgeon performing fewer robotic hernia repairs compared to laparoscopic vs. open hernia repair. However, robotic surgery coupled with 5G internet allows telesurgery to be performed, allowing the operating surgeon to operate remotely from the patient [30].

## Discussion

The use of robotic surgery enhances the possibility of repair in ventral hernia surgery which is expanding worldwide, especially with the adoption of newer techniques in hernia repair. It facilitates the shift from open surgery to a minimally invasive technique that has led to an increased percentage of adoption of endo-laparoscopic hernia repair. It has proven to have better clinical outcomes, especially in ventral hernias and complicated inguinal hernias. It facilitates the adoption of minimally invasive procedures but also facilitates surgery via extraperitoneal and retromuscular approaches that are technically demanding and difficult with the traditional endo-laparoscopic approach. Nevertheless, there is a need to evaluate the long-term outcome and cost-effectiveness of robotic hernia repair and determine if robotic hernia repair should be offered to all patients as a standard procedure. It is still a fascinating technology that requires a deep-quality study analysis by experts and academics.
